# Accuracy of Contrast-Enhanced Ultrasound for Hepatocellular Carcinoma Post-Transcatheter Arterial Embolization

**DOI:** 10.3390/jcm13247720

**Published:** 2024-12-18

**Authors:** Kathryn L. McGillen, William Watkins Pryor, Nelson S. Yee, Junjia Zhu, Karen L. Krok, Peter N. Waybill

**Affiliations:** 1 Department of Radiology, Penn State Health Milton S. Hershey Medical Center, Hershey, PA 17033, USA; pwaybill@pennstatehealth.psu.edu; 2 Department of Radiology, University of North Carolina, Chapel Hill, NC 27599, USA; will_pryor@med.unc.edu; 3 Department of Medicine, Penn State Health Milton S. Hershey Medical Center, Hershey, PA 17033, USA; nyee@pennstatehealth.psu.edu (N.S.Y.); kkrok@pennstatehealth.psu.edu (K.L.K.); 4 Public Health Sciences, Pennsylvania State University College of Medicine, Hershey, PA 17033, USA; jzhu2@pennstatehealth.psu.edu

**Keywords:** contrast ultrasound, hepatocellular carcinoma, TACE, Lumason

## Abstract

**Background/Objectives**: Contrast-enhanced ultrasound (CEUS) is a non-invasive imaging technique with similar accuracy to CT and MRI for the diagnosis of hepatocellular carcinoma (HCC). CEUS offers several advantages in patient populations who have contraindications for CT or MRI. There are limited prospective studies in the United States evaluating the diagnostic equivalence of CEUS following transcatheter arterial chemoembolization (TACE) with same-day CT/MRI. This prospective pilot study compared CEUS and CT/MRI in patients with HCC following TACE in a United States population using Lumason^®^ contrast agent and the Liver Reporting and Data System (LI-RADS). **Methods**: Following institutional review board protocols, adult patients with a diagnosis of HCC were included. Follow-up CT/MRI was directed by referring clinicians, and CEUS was performed on the same day. CEUS was used to evaluate for treated lesion(s), new lesion(s), and portal vein thrombus before and after Lumason^®^. Any subsequent follow-up imaging was reviewed. **Results**: In 26 enrolled patients, 33 target lesions were identified (size range 0.9–16.8 cm), and 26 were LI-RADS-5 or -M. CEUS identified 19 cases of residual tumor, 12 with no viable disease; CT/MRI identified 17 cases of residual tumor, 16 with no viable disease (*p* = 0.617). Both CEUS and CT/MRI identified five portal vein thrombi. Two lesions were missed or miscategorized on CEUS, while six were missed or miscategorized on CT/MRI (*p* = 0.289). Six new lesions were identified on both CEUS and CT/MRI. Of these new lesions, four were identified only by CT/MRI and three only by CEUS. **Conclusions**: CEUS is comparable to CT/MRI performed at identical follow-up intervals in evaluating for residual versus treated HCC following first-time TACE.

## 1. Introduction

Contrast-enhanced ultrasound (CEUS) has been in use globally for decades, but it has only more recently been approved for clinical use in the United States. The accuracy of CEUS is similar to MRI and CT scans in establishing the non-invasive diagnosis of hepatocellular carcinoma (HCC) in at-risk patients [1,2], with guidelines established for non-invasive diagnosis by the American College of Radiology [3]. Worldwide, HCC is at least the sixth most common tumor overall and, in terms of mortality, is ranked third [1]. Globally, hepatitis B virus infection is one of the highest causative factors, but in the United States, additional etiologies, including alcohol-related cirrhosis, hepatic C virus infection, and metabolic dysfunction-associated steatohepatitis (MASH), are common [1,2].

Several treatments are available for HCC, including surgery, radiofrequency ablation, local palliative radiation, and Yttrium-90 radioembolization, but one of the most common is transcatheter arterial chemoembolization (TACE). This treatment is used in patients who may not be candidates for curative resection (in general, less than 20% are) [4] and/or as a bridge to curative liver transplant and results in focal tumoral necrosis through catheter delivery of embolic and/or chemotherapeutic agents through the tumor hepatic arterial supply. Generally, four-phase CT or dynamic MRI are performed 4–6 weeks after treatment to determine if the tumor is completely or partially treated, based on the recommendations of the Society of Interventional Radiology [4]. This time was established to allow the differentiation of peripheral viable tumors from inflammatory peritumoral infiltration since the elimination of lipiodol by normal Kupffer cell-mediated phagocytosis requires 3 to 4 weeks [5]. Ethiodized oil uptake on CT is indicative of tumor necrosis, and multiphase CT and MRI both evaluate for cessation of previously present arterial enhancement to confirm treated HCC [4].

In general, CEUS has many benefits over CT and MRI, including a lack of radiation, the IV contrast is not nephrotoxic, overall decreased cost, and increased accessibility—both availability of appointments and patient tolerance to the examination (i.e., breath-holds, claustrophobia) [2,6]. In ultrasound, a patient’s preference for semi-upright imaging often can be accommodated, and ultrasound probe positioning may be able to track a lesion in the field of view even if the patient cannot hold their breath well, neither of which is diagnostically possible in CT or MRI. Intrinsically, ultrasound has high spatial and temporal resolution and has the potential for identifying residual or recurrent tumors at the same rates and potentially even earlier than a CT scan, where the contrast agent Ethiodol^®^ (ethiodized oil) can obscure subtle signs of residual tumor [7]. CEUS may also be useful in patients with other contrast allergies, difficulty tolerating MRI or CT, or intrinsic hardware that could affect diagnostic imaging. Because the contrast agents in CEUS are purely intravascular microbubbles and do not diffuse into the parenchyma, CEUS can re-dose within a few minutes, whereas repeat CT or MRI imaging with contrast are not possible in that time frame if not initially diagnostic [5]. However, neither ultrasound nor CEUS are an established part of the post-treatment imaging algorithm—multiphase CT and MRI are the mainstay in evaluation for successfully treated HCC. While CEUS has been established as accurate in the initial diagnosis of HCC [1,2], if also able to accurately diagnose treatment success, it would provide an additional imaging option to hepatologists and oncologists to ensure that HCC patients are triaged appropriately and potentially decrease morbidity with a safe, accurate, and less expensive bridge to liver transplantation.

Diagnostic CEUS most often utilizes a split screen technique when performing the exam. Half of the screen is a low B-mode grayscale, which ensures that the target lesion remains in the field of view, while simultaneously, the low B-mode also decreases the rate of “popping” of the IV-injected contrast bubbles compared to a diagnostic B-mode due to its lower power. The other half of the screen is the contrast screen. Initially, it is dark except for intrinsically bright structures (such as calcification or fascial lines). The timer is started as contrast is injected, and as contrast reaches the liver, the vessels and then tissue enhance and become brighter. With CEUS and the use of real-time and/or cine clips, the radiologist can watch the target lesion enhancement characteristics over several minutes, as opposed to selected times or “screenshots” as is done in CT/MRI. The radiologist can also control how long or how frequently a target is imaged (or even re-imaged). To diagnose HCC by CEUS, the radiologist looks for arterial phase enhancement above the background liver and mild washout on the delayed phase (LI-RADS 5) [3]. However, post-TACE, the diagnosis of tumors is performed differently. Treated HCC will appear as a “black hole” in the liver parenchyma—no enhancement is seen within the treated, necrotic tumor on the contrast screen, which is in many ways similar to the subtraction phases of a post-contrast MRI. In contradistinction, residual tumors will appear as thick, nodular, or mass-like enhancement. It will often demonstrate washout on delayed phase on CEUS, but visualizing this is not necessary for the diagnosis, similar to CT. Residual HCC, especially if the tumor is large, will often have successfully treated portions (the “black holes”) intermixed with areas of enhancement, which represent the residual HCC. There is an important distinction to note when performing CEUS for this purpose, in that thin, regular enhancement at the periphery of the treated tumor is not considered a residual, untreated disease but is known as post-treatment inflammatory rim enhancement (PTIRE). The difference between this and the irregular enhancement of residual HCC can present a learning curve when starting this kind of imaging program but must be recognized and accurately determined, as PTIRE does not require repeat treatment or closer surveillance monitoring. Finally, CEUS can reliably identify tumor thrombus in the portal vein and determine whether it is bland or tumor thrombus, which significantly affects patient care and transplant eligibility. A bland thrombus will not enhance (“black hole”) because it is not vascularized, while a tumor thrombus will demonstrate internal enhancement. Occasionally, arterial-type enhancement can even be seen in the portal vein, originating from within the liver and spreading outward (hepatofugal), representing the arterial vascularization of the tumor thrombus, as opposed to normal opacification of the portal vein, which is in a hepatopedal direction, from the gastrointestinal tract and spleen into the liver.

Although there are multiple case reports demonstrating the diagnostic equivalence of CEUS with CT/MRI for detecting HCC recurrence post-TACE [8,9,10,11,12,13,14,15], until recently, there have been limited studies performed in the United States (U.S.) using the purely intravascular contrast agent Lumason^®^ ([Bracco Diagnostics], SonoVue outside of the U.S., Milan, Italy). Limitations in prior studies are small sample sizes, non-standard imaging timeframe after TACE, variable definitions of residual tumor, and some being over a decade old [9,12,15,16,17,18,19,20]. Advancements in ultrasound technology and larger prospective studies are needed to determine the feasibility of CEUS being used to diagnose HCC after treatment in differing patients.

This prospective study was performed to collect pilot data that would lead to the evaluation of non-inferiority between CEUS and the clinical gold standard of CECT/MRI in patients with HCC following TACE treatments in a U.S. population using Lumason^®^ contrast agent and the Liver Reporting and Data System (LI-RADS).

## 2. Materials and Methods

**Figure A1:**
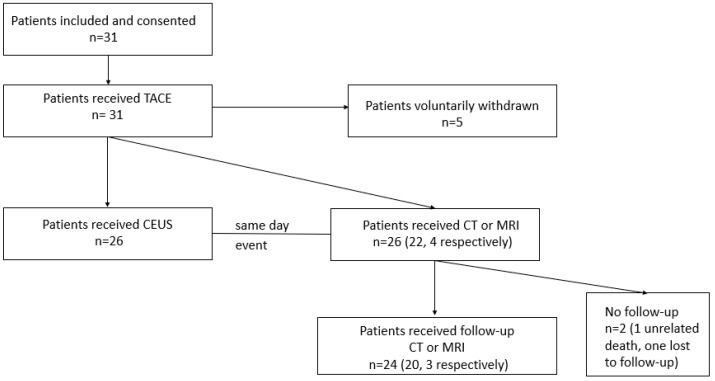
Flow chart of study patients.

This single-institution prospective study was approved by the hospital Institutional Review Board. Adult patients (>18 years of age) who received a first-time diagnosis of HCC and ≤3 lesions to be treated from December 2020 to May 2022 and were referred for TACE treatment were reviewed for enrollment (Figure A1). At our institution, all TACE orders are reviewed by a single interventional radiologist and patients were identified at that time as meeting the above requirements. Exclusion criteria were previously treated HCC, pregnant patients, pediatric patients, or patients who were unable to consent. All eligible patients were approached for consent for this study prior to their TACE. They were instructed that they could withdraw at any time from this study and that their involvement would not affect their treatment. Patients would receive a USD 50 gift card after completion of the CEUS portion of the study. Of those who consented, five patients withdrew from this study. All of these occurred after the TACE but before their follow-up imaging and the CEUS.

The hepatologist referring the patient for TACE also directed their follow-up imaging timing and modality (CT versus MRI) to evaluate for residual tumor and were blinded to the patient being enrolled in this study. During CT/MRI scheduling, patients were also scheduled for CEUS at the same visit and site as CT/MRI. The CEUS consisted of grayscale and color Doppler imaging looking for treated lesion(s) characteristics, for any new lesion(s), and for portal vein thrombus. Lumason^®^ (sulfur hexafluoride lipid-type A microspheres) was then administered intravenously (1 mL dose) to evaluate for residual tumors and to characterize any new focal findings by the radiologist (KM). The results of the CEUS were not interpreted in real time, and its results were, therefore, not available to the hepatologist, who acted only upon the results of their ordered CT/MRI.

Utilizing Research Electronic Data Capture (REDCap), a secure web application for building and managing databases, patient demographics and etiologies of cirrhosis were recorded, as were the number and size of the initial tumor diagnosed on CT/MRI.

Two radiologists (an abdominal fellowship-trained radiologist with 9 years of clinical experience and, at the time of this study, 4 years’ experience with routine CEUS indications—KM, and a radiology fellow WP, who had less than 1 year of CEUS experience at the start of this study) reviewed the initial imaging and the CEUS images. They were blinded to the same-day post-treatment CT/MRI images and report.

The presence or absence of residual tumors (LI-RADS—“treated” versus “viable”) was recorded for CEUS, as well as the grayscale echogenicity of the lesion and the presence of any limitations in seeing the target lesion(s). If new lesions were present, a LI-RADS category was assigned [3]. Portal vein patency or thrombus was recorded and, if present, characterized as bland or tumoral. A “viable” tumor after TACE was defined as arterial phase mass-like or nodular enhancement within a treated lesion. “Treated” or non-viability was defined as no enhancement within a treated lesion. Thin, regular peripheral enhancement was not considered viable but was labeled as “post-treatment inflammatory rim enhancement” and negative for tumors. Any additional findings, such as new ascites or local lymphadenopathy, were looked for and noted if present.

The official reports of the corresponding post-treatment CT or MRI were written the same day as the CEUS and were mined for the presence or absence of residual tumors, new lesions, portal vein thrombus, and evaluation for extrahepatic findings such as metastatic disease that would not be visible via ultrasound. All post-treatment CT or MRI were performed with multiple phases (pre-contrast and post-IV contrast late arterial, portal venous, and 3 min delayed phase; Omnipaque for CT, Gadavist for MRI) per departmental protocol for HCC.

If the patient returned for additional interval follow-up imaging or for treatment of residual disease, those imaging reports were mined for the above data to serve as a tiebreaker if the CEUS interpretation and same-day CT/MRI report were discordant.

### Statistical Methods

Patient demographics and main clinical characteristics were summarized using descriptive statistics. To compare the results of the post-treatment CEUS and CT/MRI, we used McNemar’s test for binary outcomes (such as whether or not the residual tumor was identified) and a paired-sample *t*-test for quantitative outcomes (such as the size of the tumor). All analyses were performed using statistical software R version 4.4.0 (R Foundation for Statistical Computing, Vienna, Austria). All tests were two-sided, and the statistical significance level used was 0.05. Due to the exploratory nature of this study, the significance level was not adjusted for multiple testing. Because of the small cohort necessitated by the pilot study funding, an initial power analysis was not able to be performed.

## 3. Results

### 3.1. Demographics

Twenty-six patients were enrolled and completed this study (19 male and 7 female) with a mean age of 66.6 years old (standard deviation of 7.9, range of 43–84) and mean body mass index of 30.5 kg/m^2^ (standard deviation of 4.9, range of 21–41). Most patients identified as white (*n* = 22, 84.6%), with the remaining patients identified as black (*n* = 2, 7.7%) or other (*n* = 2, 7.7%). The most common primary etiology of the underlying HCC risk factor in our study was hepatitis C virus infection (*n* = 14, 53.8%), followed by MASH-related liver disease (*n* = 6, 23.1%) and alcohol-associated cirrhosis (*n* = 5, 19.2%). A total of 33 target lesions were identified from the initial imaging, with sizes ranging between 0.9 cm and 16.8 cm (mean of 3 cm ± 2.8 cm); 26 of these were characterized as LI-RADS 5 or M on pre-procedural imaging. LI-RADS 4 and M lesions were definitively diagnosed as HCC by tissue biopsy prior to their referral for TACE treatment.

Time between TACE and follow-up imaging was dependent upon ordering physician preferences and then on patient availability. The range in this study was 29 to 127 days (4.1–18.1 weeks) with a mean of 66.9 days (9.6 weeks) between treatment and first imaging follow-up. The range was similar between patients with completely treated tumors and those with residual disease (the completely treated tumor follow-up range was 29–107 days).

Most patients had multiphase CT at all three time points (pre-TACE, post-TACE, and follow-up) (*n* = 15), while two had exclusively MRI, and the rest (*n* = 9) had a mix of both modalities (Table A1).

### 3.2. Post-TACE Lesion Characteristics

No patients experienced any minor or major reactions to the contrast administration.

CEUS identified 19 cases of residual tumor (19/31, 61.3%) and 12 cases with no viable disease (12/31, 38.7%). Two lesions were not visualized on follow-up CEUS, nor the follow-up CT or MRI, when compared to the pre-TACE imaging. These were considered concordant results, and the original CT/MRI was deemed an overcall of LIRADS 5. CT/MRI identified 17 cases of residual tumor (17/33, 51.5%) and 16 with no viable disease (16/33, 48.5%), *p* = 0.617. Both CEUS and CT/MRI identified the same number of portal vein thrombi (*n* = 5) with results summarized in Table 1. The sensitivity of CEUS was 94.7% and the specificity was 92.9% for correctly identifying the presence or absence of tumors.

Nine discordant lesions were present when comparing the CEUS and CT/MRI groups (Table 2). Two lesions were not visible on CEUS (6.7%) that were seen on CT/MRI. Six lesions were missed or miscategorized on CT/MRI (20%) when compared to the second follow-up CT/MRI performed as part of their standard care, which was not significantly different (*p* = 0.289). A total of six new lesions were identified on both CEUS and CT/MRI, ranging between 0.7 cm and 3.3 cm (mean 1.6 cm ± 1 cm) on CEUS and 0.7 cm and 2.8 cm (mean 1.7 cm ± 0.8 cm) on CT/MRI. However, the six new lesions on CEUS were not all the same as the six new lesions seen on CT/MRI. Four of the new lesions were identified only on CT/MRI and three only by CEUS. Two target lesions were not seen by CEUS or CT, with both originating from outside institution MRI reads.

When able to be determined, grayscale echogenicity of the lesions after TACE revealed 10 hyperechoic lesions (33.3%), while 8 were isoechoic (26.7%), 7 heterogeneous (23.3%), and 5 hypoechoic (16.7%) (Table 3). Echogenicities were similar between treated tumors and those with residual disease, except for hyperechoic lesions. Of the incompletely treated lesions, eight of these were hyperechoic, while only two completely treated tumors were hyperechoic. Despite the trend, there was no statistically significant association between pre-contrast echogenicity and residual disease (*p* = 0.2870) to predict residual versus treated disease from grayscale appearance alone.

## 4. Discussion

In this prospective pilot study (ClinicalTrials.gov ID NCT04569799), CEUS and standard of care contrast-enhanced CT/MRI were compared for accuracy in the evaluation of HCC following TACE therapy in a United States population using Lumason^®^ contrast agent. Results of this study indicate that overall accuracies are comparable between CEUS and CT/MRI with high sensitivity and specificity of CEUS in correctly identifying the presence or absence of residual tumors. All modalities identified residual tumors and portal vein thrombus with comparable accuracy. Both modalities also identified lesions that the other did not, though all but one were low-grade lesions that would be followed but otherwise not intervened upon.

Recently, a multi-institutional prospective trial evaluating CEUS accuracy after TACE using the contrast agent Definity^®^ (perflutren lipid microspheres) was reported by Savsani et al. [21]. They found that CEUS performed 4 to 6 weeks after TACE had a sensitivity higher than CT or MRI for detection of residual tumors, though with lower specificity. Though our cohort was smaller, our study also demonstrated that CEUS was comparable to CT/MRI and was useful in identifying subtle residual tumors. With that, our study had several important differences. In our study, all patients were included without preceding knowledge of whether the target HCC lesion was sonographically visible on CEUS. This may be more akin to real-world practice and allows the inclusion of isoechoic lesions, which comprised a significant 26.7% of our cohort. Additionally, our study evaluated the presence of portal venous thrombosis in CEUS compared with CT/MRI, allowed for inclusion of more than one lesion, evaluated new lesions, and CEUS was performed on the same day as the CT/MRI.

Additionally, our study included relatively small lesions. While the study by Savsani et al. does not list the average size pre-treatment, their treated lesion size was 3.8 cm, as presumed from the CT/MRI, whereas our post-treatment average size was a more diminutive 2.3 cm for target lesions. Our study, therefore, adds to the study by Savsani et al. and suggests that CEUS need not be limited to patients with only one lesion or only with larger lesions but can be used for multiple, smaller nodules and should include isoechoic lesions.

**Figure 1:**
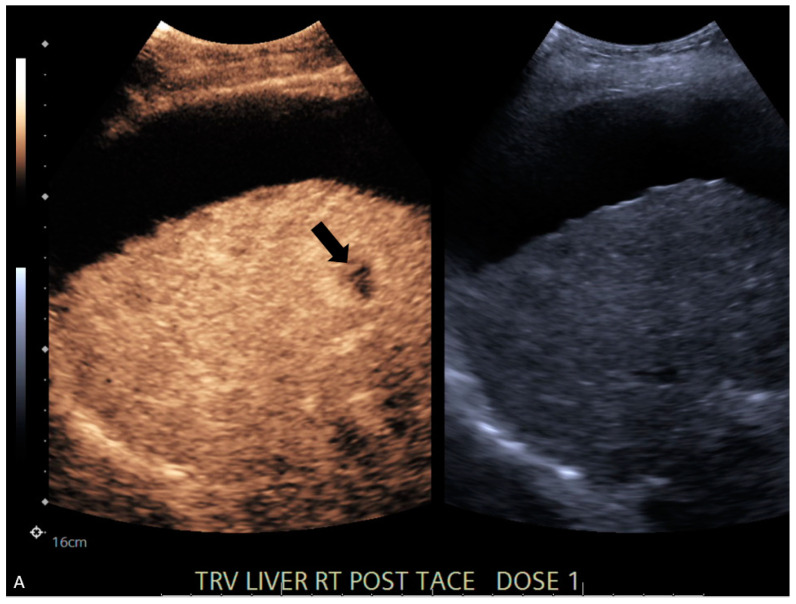

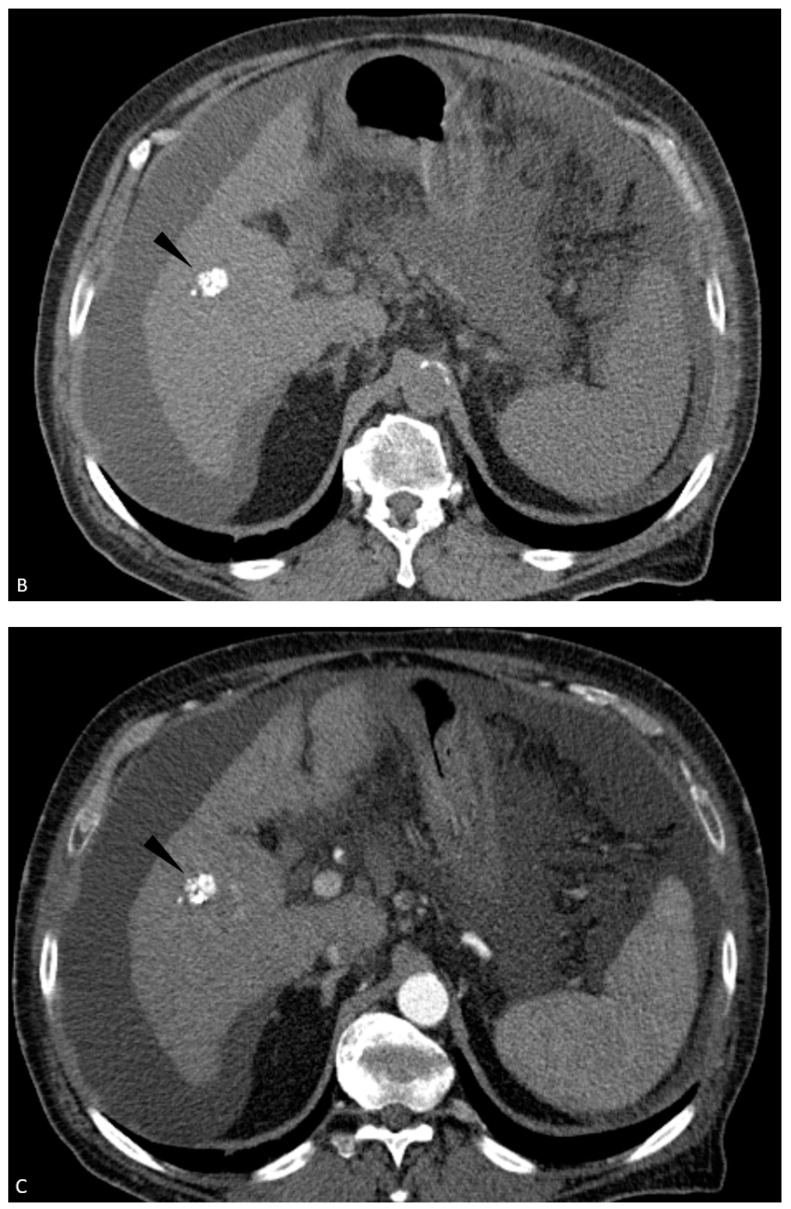
A 63-year-old male with cirrhosis due to MASH. (**A**) CEUS and accompanying grayscale image at 14 s following administration of Lumason^®^. Black arrow corresponds to internal enhancement thought to represent residual disease. Enhancing area demonstrated washout on delayed images. (**B**,**C**) Corresponding same-day CT showing no viable, enhancing lesion (black arrowheads), (**B**) non-contrast CT, (**C**) late arterial-phase CT.

In our study, some new lesions were seen only on CT/MRI, and some only on CEUS. On follow-up, only one new tumor was missed by CEUS (LR-5 on CT), whereas in two cases, CEUS had a lower LI-RADS score than CT for new lesions. For target lesions, only in one patient could the lesion not be seen on CEUS but was present and visible on CT. Limitations to CEUS are the same as those in grayscale ultrasound: small or subtle lesions may be missed if they are obscured by the lung (particularly affecting the liver dome) due to steatosis or heterogeneity of the background liver, though they may still be visible by CT or MRI [3,22]. Finally, CEUS overcalled one case as residual disease due to vague internal enhancement (Figure 1), which was not seen on initial or 3-month CT follow-up. More new lesions were visible by CT, though most were not diagnosed as HCC by imaging (i.e., were LR-3 lesions) and would have been followed. Perfusional abnormalities are often classified as LR-3 lesions on CT and may account for some of these discrepancies.

**Figure 2:**
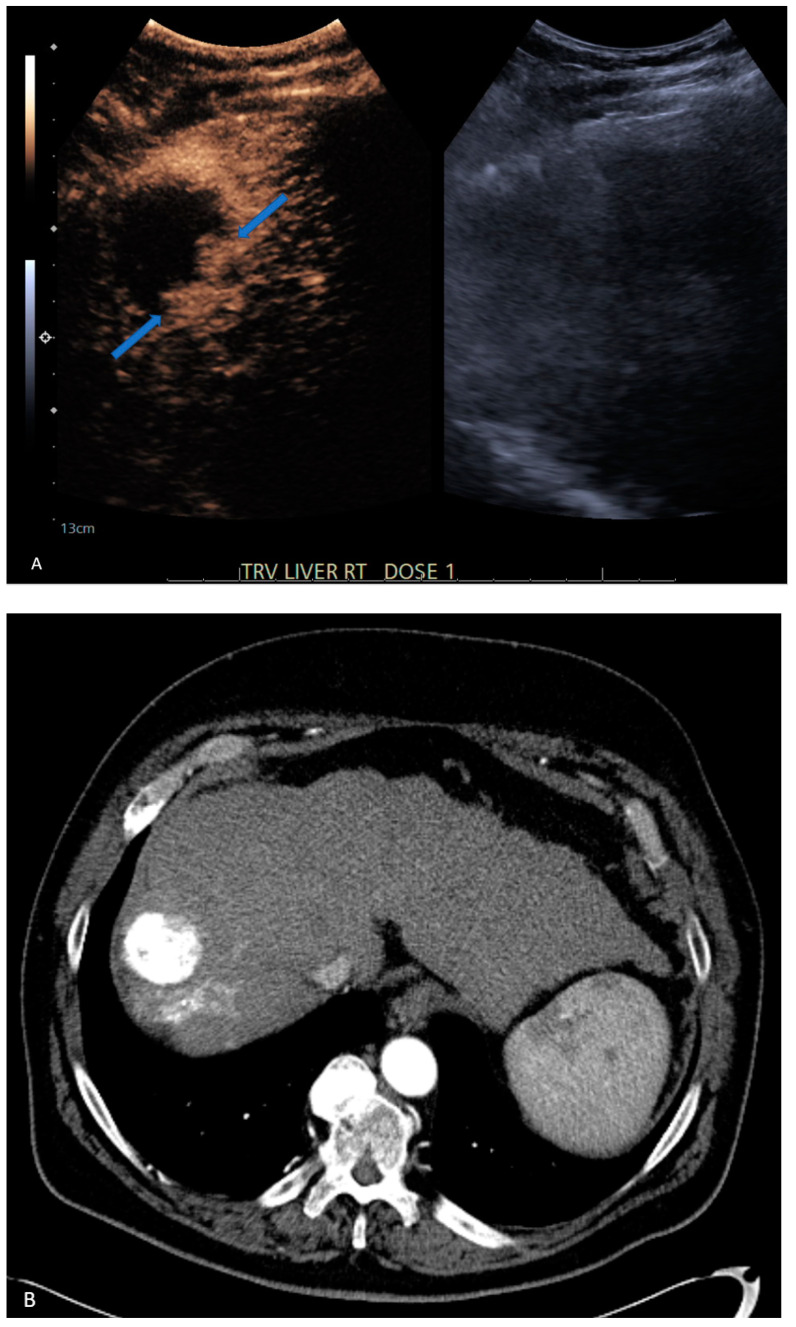

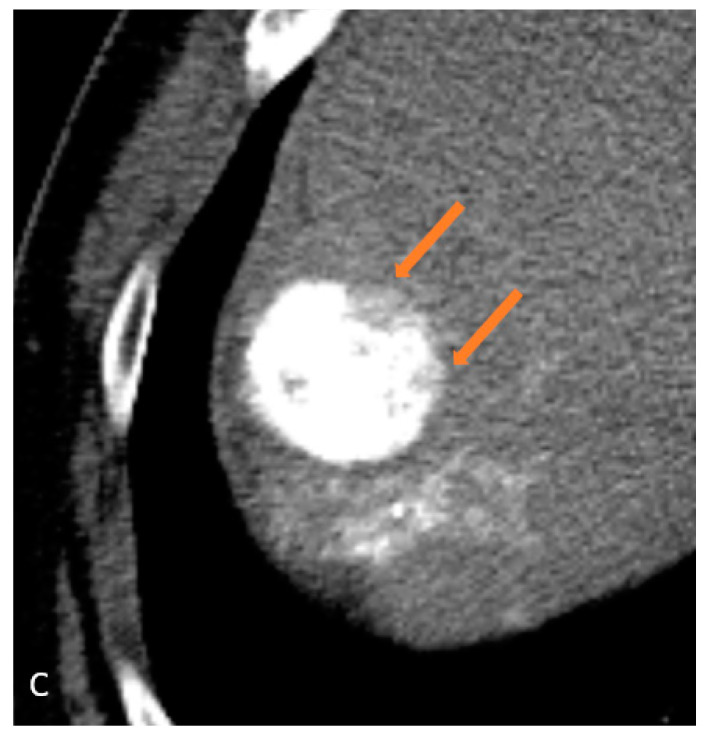
A 63-year-old male with hepatitis C-induced cirrhosis. (**A**) CEUS demonstrates two areas of focal peripheral nodular enhancement in the treated lesion (blue arrows), indicating residual disease. Initial follow-up CT (**B**) was read as non-viable lesions (LR-treated). Three-month follow-up CT confirmed CEUS findings of residual disease. Retrospective review of the initial CT demonstrated nodular regions of enhancement, better seen on enlarged view of the lesion ((**C**), orange arrows).

**Figure 3:**
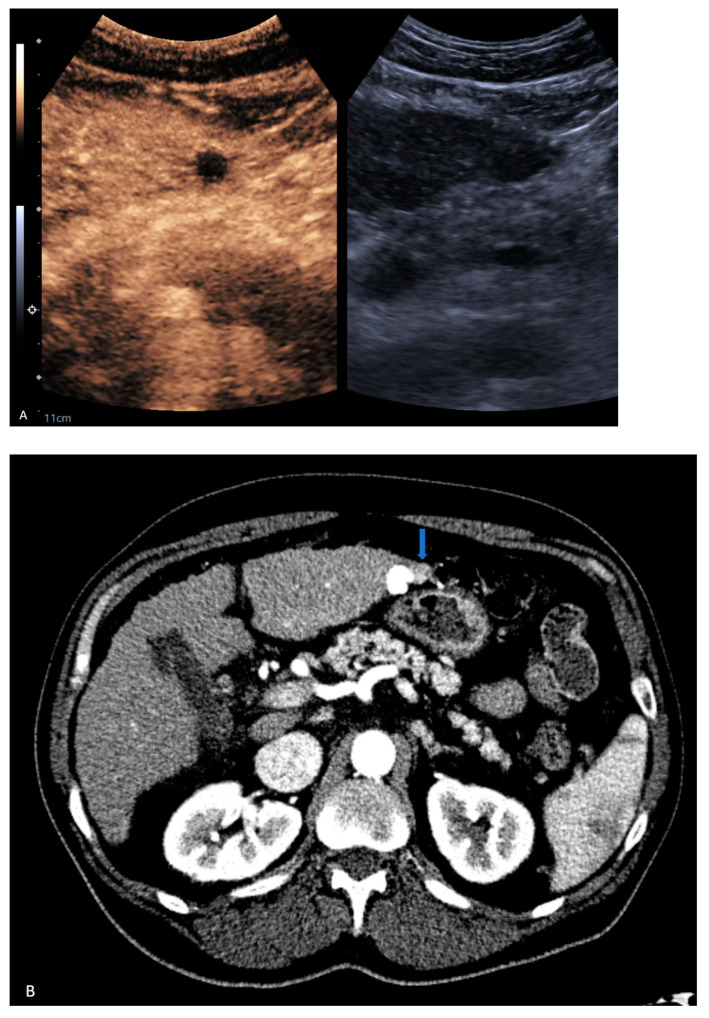
A 68-year-old male with cirrhosis secondary to MASH. (**A**) CEUS and accompanying grayscale demonstrate the completely treated lesion. Initial CT (**B**) shows region of enhancement (blue arrow) adjacent to the Ethiodol that was called residual disease. Three-month follow-up CT showed region of enhancement was perfusional and not viable tumor.

CEUS excelled in differentiating adjacent perfusional change from residual tumors (Figure 2) and small residual tumoral enhancement from completely treated disease in cases where Ethiodol^®^ presence likely masked the subtle enhancement (Figure 3). This is concordant with Savsani et al.’s findings of CEUS’ utility prior to the expected complete resorption of Ethiodol^®^ before the established 4 to 6-week timeframe. CEUS has an excellent temporal resolution in visible lesions—CT and MRI may be limited when contrast bolus timing is suboptimal, or there is patient motion, which can often be overcome in CEUS. This may also account for cases where CEUS outperformed CT/MRI.

Extrahepatic disease is unlikely to be diagnosed by CEUS unless it is locoregional and seen on grayscale evaluation. While our study did not find that CEUS missed any extrahepatic findings, the sample size was small and did not include any patient with metastatic disease at follow-up.

While not statistically significant, the pre-contrast echogenicity of treated lesions may represent an area for future investigation and provide useful information to target Doppler and microvascular imaging (when available). The pre-contrast echogenicity of treated lesions may also assist in targeting for CEUS and add diagnostic certainty to CEUS. Since HCC is often hypoechoic on initial diagnosis, it was not surprising that most of our cases with hypoechoic residual lesions represented viable residual disease. While we anticipated that echogenic lesions may represent successfully treated lesions given the presumed presence of retained Ethiodol^®^ [23], we instead found more cases of viable tumors were present than not in hyperechoic lesions (Table 3). Lastly, isoechoic residual disease is the most difficult to detect on follow-up grayscale ultrasound, necessitating a firm understanding of where the initial disease was presented on pretreatment images, as 16% of cases were mostly isoechoic yet still viable tumors.

### Limitations

**Figure 4:**
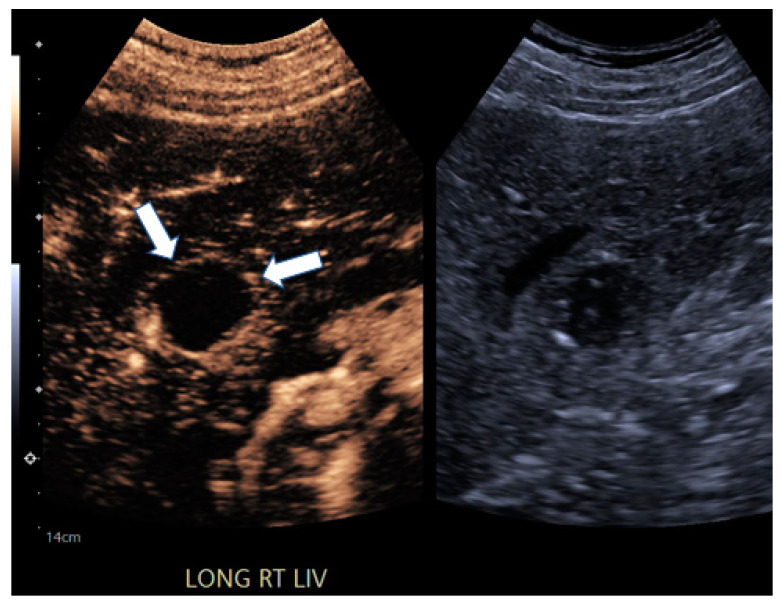
A 69-year-old male with hepatitis C-induced cirrhosis. CEUS image taken 19 s after contrast administration demonstrates thin, peripheral enhancement (white arrows), which is consistent with post-treatment inflammatory rim enhancement (PTIRE) and is not representative of residual HCC.

There were several important limitations to this pilot study, including the limited statistical power associated with a small sample size. For clinical adoption, there is a learning curve associated with the interpretation of post-TACE CEUS images, specifically PTIRE, which, until recognized, can look similar to residual disease. Our readers batch read the CEUS and so gained the needed experience to be able to reliably recognize the difference (Figure 4). While it is straightforward to identify the treated tumor black holes, it is initially more challenging to recognize and differentiate subtle residual tumors versus the post-inflammatory, smooth peripheral enhancement that is not infrequently seen. Future studies are needed with a larger sample size to confirm results and to better identify specific populations that may have the most accurate CEUS results, such as sorting via the Visualization category in ultrasound LI-RADS [3] or by lesion size or echogenicity.

## 5. Conclusions

The accuracy of CEUS in our study was comparable with CT/MRI for evaluating residual HCC following TACE and for portal vein thrombus, with high sensitivity and specificity in CEUS. While this was a small pilot study, conclusions are limited, but it indicates the potential of CEUS. However, larger, multi-institutional studies are needed to confirm accuracy and to be considered as an option for patients with other contrast allergies or who have contraindications to MRI. CT and MRI are the gold standards, but further studies are needed to determine if CEUS may be beneficial in patients who require limited imaging for tumor viability or recurrence, who have a prognostically shortened lifespan, or who are awaiting transplants. As CEUS is inherently limited in evaluation for extrahepatic metastatic disease, it may be best used in the above circumstances or for cases where CT or MRI gave unclear or indeterminate results. This early, small study suggests that CEUS may be considered as an alternate modality in patients where the lesion is isoechoic and small and in cases with more than one lesion. Further larger prospective trials are needed to draw more definitive conclusions.

## Figures and Tables

**Table 1:** Summary of lesion observations post-TACE for both modalities. Thirty-three pre-treatment lesions were present, 2 of which were not visible by either CEUS or CT/MRI.

	LI-RADS Treated	LI-RADS Viable	New Lesion	Portal Vein Thrombus
CEUS	12 (38.7%)	19 (61.3%)	6	5
CT/MRI	16 (48.5%)	17 (51.5%)	6	5

**Table 2:** Discordant lesion summary (*n* = 9). Correct modality was determined by follow-up imaging results (persistence or absence of enhancement of the questioned lesion). Two lesions noted as discordant are compared to the initial pre-imaging (8 and 9), otherwise, discordancy was between follow-up imaging.

Subject	Correct Modality	Target Lesion or New Lesion	LI-RADS	Additional Information
1	CT	New	LR-4	CEUS showed new lesion LR-3. Follow-up CT the lesion progressed to LR-5
2	CT	New	LR-5	US did not identify new lesion
3	CT	New	LR-3	US did not see new lesion, progressed to LR-4 on follow-up
4	CT	Target	LR-treated	CEUS called residual tumor (Figure 1), follow-up CT confirmed treated tumor
5	CT	Target	LR-treated	US was unable to see the target tumor at all
6	CEUS	Target	LR-viable	CT missed subtle disease at the periphery of the treated HCC (Figure 2)
7	CEUS	Target	LR-treated	CT called residual tumor, follow-up CT showed the area was perfusional, not disease (Figure 3)
8	Other	Target	Not seen	One of 2 target lesions was not seen on CEUS, CT, or follow-up CT
9	Other	Target	Not seen	One of 2 target lesions was not seen on CEUS, CT, or follow-up CT

**Table 3:** Echogenicity of target lesions after TACE treatment as observed on grayscale ultrasound, prior to contrast administration.

Grayscale Echogenicity of Tumor After TACE	LIRADS—Viable (*n* = 19)	LIRADS—Treated (*n* = 11)
Hypoechoic	4 (21%)	1 (9%)
Isoechoic	3 (16%)	5 (45%)
Hyperechoic	8 (42%)	2 (18%)
Heterogeneous (mixed)	4 (21%)	3 (27%)

## Data Availability

The data presented in this study are available upon request from the corresponding author due to privacy and legal reasons.

## Appendix B

**Table A1:** Imaging modality of diagnosis. One patient had follow-up via TACE only with no further imaging at the institution, 2 patients had no additional follow-up at the institution after their initial post-treatment imaging, which are denoted as N/A, not available.

	Pre-TACE	Post-TACE	Follow-Up
1	CT	CT	CT
2	CT	CT	CT
4	CT	CT	CT
5	CT	CT	CT
6	CT	CT	CT
7	MR	CT	CT
8	MR	MR	N/A
9	CT	CT	CT
10	CT	CT	TACE
11	CT	CT	CT
12	MR	MR	CT
14	CT	CT	CT
15	CT	CT	MR
18	CT	CT	CT
19	CT	CT	CT
20	CT	CT	CT
21	CT	CT	CT
22	MR	MR	MR
23	CT	CT	CT
24	CT	CT	CT
25	MR	CT	CT
26	CT	CT	CT
27	MR	MR	MR
28	MR	CT	CT
30	MR	CT	CT
32	MR	CT	N/A
CT	17	22	20
MR	9	4	3
